# Arsenic Release from Various Sizes of Excavated Crushed Rock Mixed with Soil at Various Ratios under Three Mass Water Contents for Cropland

**DOI:** 10.3390/toxics11030267

**Published:** 2023-03-14

**Authors:** Kyo-Suk Lee, Gil-Yong Suh, Doug-Young Chung

**Affiliations:** 1 Institute of Agriculture Science, Chungnam National University, Daejeon 34134, Republic of Korea; 2 Department of Bio-Environmental Chemistry, College of Agriculture and Life Sciences, Chungnam National University, Daejeon 34134, Republic of Korea

**Keywords:** excavated crushed rock, arsenopyrite, arsenic, dissolution, soil

## Abstract

The purpose of this experiment was to investigate the feasibility of treating arsenopyrite-containing excavated crushed rock (ECR) in cropland by examining the amounts of arsenic released from various sizes of ECR mixed with soils at different ratios under three water levels using a batch incubation experiment. A total of 4 particle sizes of ECR were mixed with soil from 0% to 100% in 25% increments under three mass water contents such as 15%, 27%, and saturation. The results showed that the amount of As released from ECR mixed with soil was in the order of 27% saturation and 15% for 180 days regardless of the ECR:soil ratios, and the increase in the amount of As released before 90 days was slightly greater than that after 90 days. The maximum and minimum contents of released As were observed at 350.3 mg·kg^−1^ (ECR:Soil = 100:0, ECR size = 0.0–0.053 mm, and Ɵ_m_ = 32.2%), indicating that the smaller the ECR particle size resulted in a higher extractable As concentration. The amount of As released was higher than the relevant standard (25 mg·kg^−1^), except for ECR with a mixing ratio (25:75) and particle size (4.75–10.0 mm). In conclusion, we assumed that the amount of As released from ECR was influenced by the higher surface area of smaller ECR particle sizes and mass water content, which determine the porosity of the soil. However, further studies are needed on the transport and adsorption of released As depending on the physical and hydrological properties of the soil to determine the size and incorporation rate of ECR into the soil in view of the government standard.

## 1. Introduction

Tunnel construction through the mountain chains generates large quantities of excavated crushed rock (ECR), which contains approximately up to 2% arsenopyrite, an arsenic-bearing sulfide mineral, resulting in the fact that the handling and treatment of ECR is a fundamental subject in a tunnel construction project. The prior risk assessment of ECR about soil contamination should be completed with respect to the concerned (25 mg·kg^−1^) and the measurable (75 mg·kg^−1^) government standards for arsenic (As) for zone 1, including croplands in Korea, because the local government authorities planned to recycle the ECR in croplands.

Under the conditions prevailing at the Earth’s subsurface, sulfide minerals, including arsenopyrite slowly convert into iron arsenates when arsenopyrite, becomes exposed to the atmosphere [1], and inorganic arsenic, which is progressively released as pentavalent arsenate (As^5+^) or trivalent arsenite (As^3+^) species [2], can exacerbate arsenic contamination in groundwater and soils [3]. The presence of arsenic in soil and groundwater is generally caused by the dissolution of As-bearing sulfide minerals (AsSM) triggered by dynamic redox conditions where atmospheric oxygen and/or oxic water contact the reduced reactive minerals [4,5]. Once released into the environment, arsenic, which can be further dispersed in the environment, may adversely affect the growth of plants and animals, becoming potential environmental and bioavailability hazards for sustainable agriculture practices and food security [6]. Consequently, there is an increasing public concern about As contamination in soil and groundwater derived from arsenopyrite, which has important implications for the prevention and control of As pollution in cropland.

A higher level of complexity is expected under cropland conditions as a result of the large number of spatially and temporally varying chemical processes that can affect the mineral transformation of As-bearing sulfide minerals [7,8]. In particular, multiple oxidative dissolution pathways of sulfide minerals have been identified via the reduction of oxygen, ferric iron, and/or nitrate [9,10,11,12]. The release of As from arsenopyrite is also affected by the pH and ionic strength, as these hydrochemical parameters exert primary control on a wide range of geochemical processes, including mineral dissolution and precipitation [13,14,15]. Most of the experimental studies of As release from AsSM have been limited to relatively simple hydrochemical conditions [3,8,16] and to the subsequent release of arsenic in solution under controlled laboratory conditions [11,17].

There will be so many variables on the release of As from ECR incorporated in cropland. The typical soil physical properties of cropland consist of bulk density and soil particle distribution, which control the porosity and water-holding capacity of the soil. In particular, soil water potential controls the soil water content ranging from the permanent wilting point to saturation, which determines the volume of the air-filled pore. Water content and corresponding air-filled volume are influencing factors on the redox potential of soils, which determine the As release from AsSM. The soil water content of cropland varied throughout the year in Korea. The climatic conditions are divided into three distinct seasons, such as the winter season from mid-November to mid-March, the drought season from mid-March to mid-June and from mid-October to late November, and the rainy season from mid-June to late July. According to the seasonal climatic conditions, the water content in the soil of cropland ranges from close to the initial wilting point (drought season) to almost saturation (rainy season). The water content for the rest of the season is below or just above field capacity. The air-filled pore volume can be changed depending on the variation in soil water content. Additionally, the pH of the soil for the cropland is generally between 6.0 and 6.5, except for a bit higher pH of 6.5–7.0 in the barley-growing field during winter. The possibility of ECR incorporation in the soils of croplands can be determined by the government’s soil pollution standards of As. Extensive research has been devoted to quantifying the dissolution rates of arsenic from arsenopyrite as a function of the size and degree of oxidation of its particles in soils. Previous research [11] indicates that grain sizes can exert significant control over oxidation and dissolution rates. McKibben et al. [11] determined that 150–250 μm was the most convenient grain size for arsenopyrite dissolution. The incorporated particle size of ECR is important in terms of the soil’s physical and chemical properties for cropland because it affects the water-holding capacity and CEC, which are factors in soil quality. However, little research has been done on the levels of arsenic release from the rocks containing arsenopyrite that are incorporated into the soils of croplands. Therefore, it is necessary to verify the levels of arsenic released from different sizes of ECR incorporated into soils, taking into account the variable climatic conditions of the field. Apart from these experiments, it needs further subsequent investigations of the fate, transport, and bioavailability of As released from ECR in soils of cropland depending on particle sizes and their relative incorporated proportion of ECR to be recycled in croplands in terms of soil physical and chemical properties.

The aim of this study was to investigate the treatment feasibility of ECR containing arsenopyrite in croplands by examining the extent of arsenic released from various sizes of ECR mixed with soils at several ratios under three levels of mass water contents (Ɵ_m_) which can be generally observed in cropland growing crops using a batch incubation experiment. Then, the results can be used to determine the possibility of ECR incorporation into croplands and the relative size and proportion of ECR incorporated into croplands in view of government standards for soil contamination of As.

## 2. Materials and Methods

The 200 kg ECR that was collected from the tunnel construction site located at Sukgye in Kyoungsangbookdo (province) was dried for 48 h at 105 °C in a hot air oven. The ECR particles that were passed through an ASTM #18 sieve (10 mm) were used in this experiment. Then, the ECR particles were divided into 4 grades of particle size using a set stack of 4 ASTM standard sieves (0–0.053, 0.053–2.0, 2.0–4.75, and 4.75–10.0 mm) in a vibratory sieve shaker (Analysette 3Pro, Fritsch, Idar-Oberstein, Germany). Oxidized species on the surface of ECR particles were removed by the application of 6N HCl according to the method suggested by Parthasarathy et al. [18]. The identification of the crystalline phase ECR particle was conducted by an X-ray diffractometer (Rigaku DMAX-2500, Tokyo, Japan) equipped with a CuKa-radiation source before and after heating and for magnesium-saturated (Mg-clay) with or without glycation. The chemical composition of each ECR particle grade was determined by a wavelength-dispersive X-ray fluorescence spectrometer (NEX QC, Rigaku, Tokyo, Japan). The properties of ECR used in this experiment are summarized in Table 1.

Soil samples were collected from the Ap horizon of cropland that had cultivated soybeans and/or corn close to the tunnel construction site. Soil samples were air-dried and ground to pass through a 2 mm sieve, stored for subsequent analysis and experiments. Soil texture and organic matter content were determined by the hydrometer method and the Walkley–Black method, respectively. pH and EC of soil were measured in a 1:5 soil-to-deionized water ratio. The extractable acids and basic cations were obtained by titration with 1.0 M KCl extract and sequential washing with ethanol and 1.0 M ammonium acetate solution, respectively. The extracted exchangeable acids and basic cations were determined by inductively coupled plasma (ICP-OES Agilent Technology 720, USA). All measurements were repeated in triplicate. The properties of the soil and rock fragment used in this experiment are summarized in Table 1. The soil particle distribution of various sizes of ECR mixed with soil at four ratios was obtained by calculating the measured results of soil and each ECR particle grade (Table 2).

### 2.1. Arsenic Release Experiment

Aerobic incubation experiments were conducted to determine the content of arsenic released from four grades of ECR particles mixed with soil in the ratio from 0% to 100% with 25% increments at three levels of mass water content (15%, 27%, and saturation) at a constant room temperature of 25 ± 1 °C for 180 days. Prior to the As release experiment, extraction of soluble and exchangeable arsenic from all ECR samples and soil was performed by sequential flow-through displacement in the following order: 5% H_3_PO_4_, distilled water, 0.005 M oxalic acid, deionized water, 0.1 M ammonium acetate, and deionized water in a round acrylic column. After this process, the amount of As in each ECR particle grade and soil was determined using the same analytical procedures described above. Each ECR sample and soil were then oven-dried, and the soil sample was ground to pass through a 2 mm sieve for the As release experiment. A total of 300 g of ECR sample mixed with soil at 4 ratios was uniformly packed by tapping around the containers using a small mallet in a 500-mL amber glass container with a nylon mesh lid. The four ECR to soil ratios were 100:0, 75:25, 50:50, and 25:75 on an oven-dried weight basis. The volume of each ECR sample was measured to calculate the bulk density (BD) and porosity (P). P was calculated by the equation as follows: P = 1 − BD/particle density (Table 3). The particle densities of ECR and soil were 2.71 g cm^−3^ and 2.65 g cm^−3^, respectively.

The calculated P was used as the saturated volumetric water content of the respective particle grade of the ECR sample. Then, the mass water content (Ɵ_m_) at saturation was obtained by dividing porosity by BD. The amount of water for each mass’s water content was calculated by multiplying the respective Ɵ_m_ by the oven-dry mass (300 g) of the respective ECR sample. The amount of water needed to saturate each ECR sample was obtained by multiplying P by the volume of each ECR sample (Table 4). The three levels of mass water content of each ECR sample were adjusted with deionized water by thoroughly mixing the sample with a spatula. ECR samples were incubated at 25 °C in a ventilated incubator for 180 days. Volumes of water and the corresponding air-filled pore for ECR are mixed with soil at four different ratios under three levels of mass water content (Table 5). pH, EC, and arsenic content of four grades of ECR variously mixed with soil are summarized in Table 6.

Using a round thin core sampler, approximately 5 g of wet ECR sample was collected from each container at sampling times of 0, 1, 2, 3, 4, 5, 7, 10, 15, 20, 30, 45, 60, 75, 90, 120, 150, and 180 days, respectively, for analysis of released arsenic. The wet ECR samples were weighed and oven-dried, and three grams of oven-dried ECR samples were then collected for the analysis of released arsenic. Extraction of released arsenic from an oven-dried ECR sample was performed by adding 60 mL of extractant (50% of 5% H_3_PO and 50% of 1 M ammonium oxalate, *v*·*v*^−1^) to three grams of ECR sample in a 100 mL plastic container. The suspension was shaken on a reciprocal shaker for 16 h and then centrifuged for 15 min at 1650 rpm. The supernatant solution was filtered through Whatman No. 40 filter paper for analysis of arsenic. The concentration of As in the supernatant solution was determined with inductively coupled plasma mass spectrometry (ICP-MS: Elan DRCe; PerkinElmer, Waltham, MA, USA) and inductively coupled plasma optical emission spectrometry (ICP-OES: Agilent 700 Series; Agilent Technologies, Santa Clara, CA, USA), respectively. The moisture content of each sample was periodically monitored and adjusted with deionized water by gently spraying and thoroughly mixing to bring the sample to the appropriate water content.

### 2.2. Data Analysis

The data obtained were statistically analyzed using R software (vECRion 3.2.2), including EZR (vECRion 1.31), on R Commander (vECRion 2.2-3). Correlation analyses among dissolved As, water content, and pH were carried out by Spearman’s rank correlation test.

## 3. Results and Discussion

The physical and chemical properties of different sizes of ECR and soil are presented in Table 1 and Table 2. The soil texture of the soil samples used in this experiment was loam, and the soil particle distribution of ECR less than 0.053 mm in diameter showed 13.1% clay and 86.9% silt, respectively. The particle sizes of ECR between 0.053 mm and 2.0 mm belonged to the silt fraction, while the particle sizes of ECR greater than 2.0 mm belonged to the gravel fraction, which is not included in determining the soil particle distribution. Therefore, the incorporation of ECR greater than 2.0 mm does not affect the soil texture, while the incorporation of ECR less than 0.053 mm may affect the proportion of clay and silt in the ECR mixed with soil. Additionally, the incorporation of larger sizes of ECR may increase the proportion of larger pores that can accelerate the water flow in soils (Table 2).

For the mineral composition of ECR, XRD results showed that the major minerals were calcite, scorodite, garnet, and magnetite, and others, including As-bearing minerals, were arsenopyrite, pyrite, niccolite, cobaltite, and claudetite. Especially the very small proportions of magnetite and amphibole as others were observed in ECR less than 2 mm (Table 7). The proportions of calcite and scorodite in ECR increased with increasing ECR particle size, while those of garnet and magnetite decreased with increasing ECR particle size. The proportion of arsenopyrite decreased from 1.28% (0.0–0.0053 mm) to 0.93% (4.75–10.0 mm).

The content of mineral-forming elements in ECR associated with the major three minerals such as calcite, scorodite, and garnet revealed that Ca, Si, and Fe were the dominant elements, and the rest of the elements occupied approximately 10%. The content of CaO increased with an increasing particle size in ECR, while the contents of Si, Fe, Mg, and As decreased with the increasing particle size in ECR. Thus, the observed variability in concentrations of the main mineral-forming elements, including Mg and As, in ECR could be influenced by its particle size, as seen in Table 8.

Arsenopyrite, which is the most dominant arsenic mineral in most As-bearing natural occurrences and intimately intergrown with pyrite, and minor amounts of other sulfides, has traditionally been considered to be chemically unstable in the surficial environment. The preliminary analysis of the chemical composition of arsenopyrite contained in ECR showed that arsenic was the dominant element at 45.6% (weight basis) and 32.5% (atomic basis) (Table 9). Based on the content of arsenopyrite ranging from 0.93% (ECR size, 7.38 mm) to 1.28% (ECR size, 0.027 mm), as seen in Table 5, the average arsenic content of the four particle grades of ECR ranged from 3023 mg·kg^−1^ to 3563 mg·kg^−1^ with decrease in the average particle size of ECR from 0.027 mm to 7.38 mm. This indicated that the contents of As in all four particle grades of ECR exceed the government standards for arsenic for the concerned (25 mg·kg^−1^) and the measurable (75 mg·kg^−1^) about soil contamination in zone 1, which includes croplands in Korea. After sequential extraction of As from the ECR sample in Table 4, the amount of indigenous As retained in the ECR particle showed that As content decreased with increasing particle size. As in ECR, it could remain as a crystalline component or adsorbed phase, which can be displaced by competitive anions in soil solution. As, which remained as an adsorbed phase, could be increased with increasing surface area and could be displaced by extractant, resulting in lower indigenous As content with decreasing ECR particle size.

The chemistry of the water and rocks indicates that arsenopyrite oxidation, which releases both S and As, could be the source of the arsenic in the water and soil because the water in contact with the arsenopyrite causes some decomposition and dissolution of arsenic, at a wide range of concentrations [19]. Field evidence demonstrated that arsenopyrite did not readily decompose under water-saturated near-surface conditions [20]. An air exposure of AsSMs, in which arsenopyrite becomes extremely soluble immediately on the oxidized side of the stability limits, reacted rapidly, and the oxidation of As to As(III) was more rapid [19].

The porosity of ECR samples can be altered by the particle size distribution of ECR and its mixing ratios with soil, resulting in changes in air- and water-filled pore volumes, as observed in Table 3. Generally, an increase in BD and the proportion of sand particles resulted in a decrease in P. Air-filled porosity can be decreased with increased water content, regardless of BD and the proportion of sand particles. As observed by Yang et al. [21], an increase in the water content of soil lowered the redox potentials in the soils except in the sandy loam soil [20], and the Eh of soil was significantly decreased as the water content increased from 24% to approximate saturation [22]. Therefore, As release from ECR containing arsenopyrite can be determined by the oxidation status depending on the water content and corresponding air content of ECR mixed with soil. In addition to the effect of the contents of water and air on the release of As from arsenopyrite, very small changes in redox potential result in large changes in the theoretical solubility of As for any given pH of soil. As release decreased with increasing pH from 5.0 to 9.0 [20]. In Table 3, the pHs of ECR and soil were 7.68 and 5.95, respectively, and pH decreased from 7.68 (0–0.053 mm) to 7.14 (4.75–10.0 mm) as the average particle size of ECR increased from 0.027 mm to 8.32 mm. Thus, the amount of As released from ECR can be governed mainly by the particle size, depending on the proportion of air- and water-filled pore volumes and soil pH.

Table 3 shows the average bulk density and calculated porosity of ECR mixed with soil at four different ratios. The volume of ECR mixed with soil decreased with an increasing particle size within the same mixing ratio but increased with a decreased mixing ratio of ECR within the same particle size range. The relative bulk density of ECR mixed with soil increased with an increased particle size within the same mixing ratio and decreased with a decreased mixing ratio of ECR within the same particle size range. The calculated porosity ranged from 0.458 (100:0 and 4.75–10.0 mm) and 0.501 (25:75 and 0–0.0053 mm), showing the difference was 0.043. This indicated that the amount of mass water content could be varied by the mixing ratio and size of the ECR. Based on the measurement of bulk density and porosity for all ECR samples, volumes of water- and air-filled pores for three levels of the mass water content in ECR samples showed that the volume of air-filled pore decreased with increased mass water content for the same ECR:soil mixing ratio while the volume of the air-filled pore increased with a decreased ECR:soil mixing ratio. The difference between maximum and minimum was greater in 27% Ɵ_m_ than that of 15% Ɵ_m_, representing that increase in Ɵ_m_ creates the more air-filled pore that can result in large changes in theoretical solubility for any given pH. Arsenopyrite does not readily decompose under water-saturated near-surface conditions, whereas Arsenopyrite becomes extremely soluble immediately on the oxidized side of the stability boundaries [23]. Therefore, an increase in air-filled pore volume can result in the dissolution of arsenopyrite from ECR.

**Figure 1:**
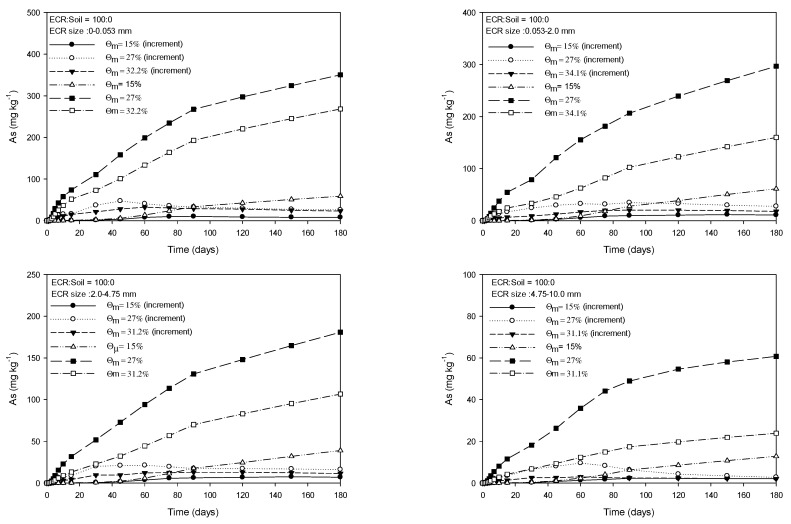
Amount of extractable As from four grades of ECR particle mixed with soil at the ratio of 100:0 under 3 mass water contents for 180 days.

**Figure 2:**
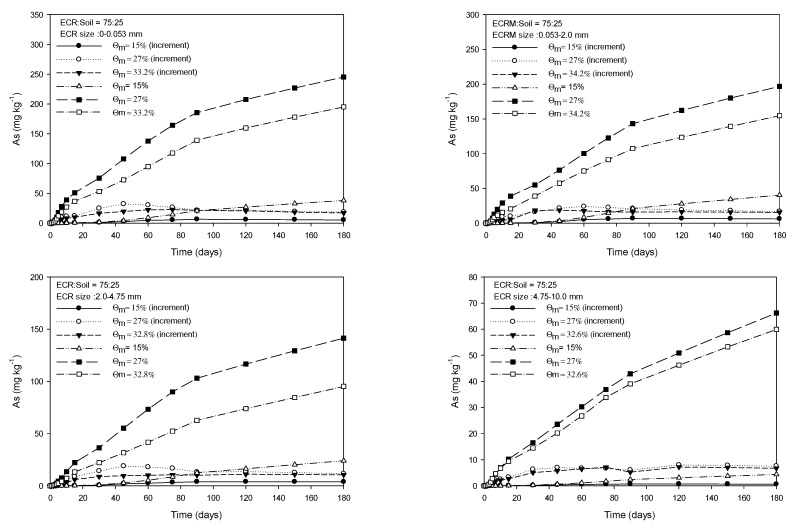
Amount of extractable As release from four grades of ECR particle mixed with soil at the ratio of 75:25 under 3 mass water contents for 180 days.

**Figure 3:**
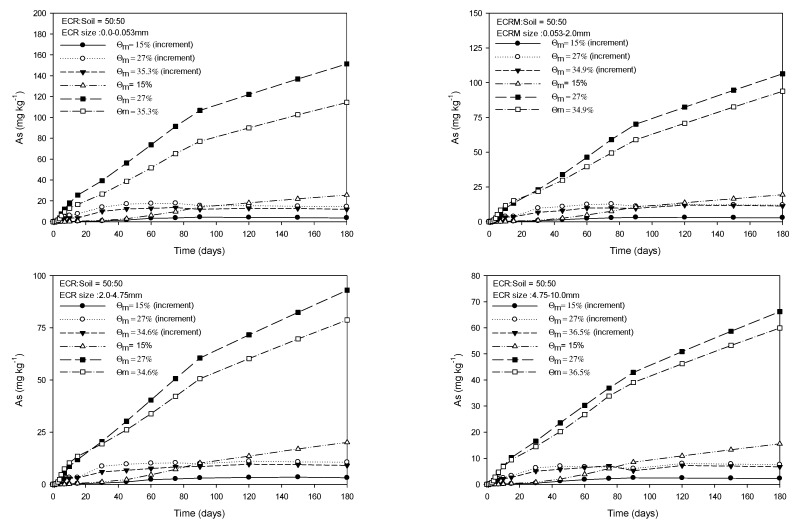
Amount of extractable As from four grades of ECR particle mixed with soil at the ratio of 50:50 under 3 mass water contents for 180 days.

**Figure 4:**
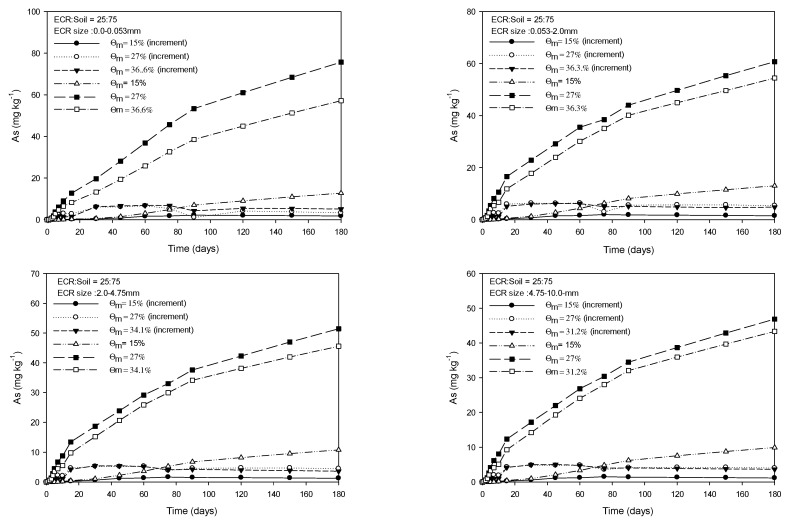
Amount of extractable As from four grades of ECR particle mixed with soil at the ratio of 25:75 under 3 mass water contents for 180 days.

Figure 1, Figure 2, Figure 3 and Figure 4 show the As released from four particle groups of ECR mixed with soils at four different ratios under three mass water contents. The average mass water content (Ɵ_m_) at saturation increased with decreasing proportions of ECR mixed with soil for the same ECR particle size, while it decreased with increasing particle size for the same ECR and soil mixing ratios. As release patterns of all four grades of ECR, which were mixed with soil at various ratios, showed that the amount of As released from ESR was in the order of 27%, saturation, and 15% of Ɵ_m_ and gradually increased until 90 days and then the increase of released As was slightly delayed for 180 days (Figure 1, Figure 2, Figure 3 and Figure 4). The maximum and minimum contents of released As were observed from 350.3 mg·kg^−1^ (ECR:Soil = 100:0, ECR size = 0.0–0.053 mm, and Ɵ_m_ = 32.2%) in Fig, 1 to 4.41 mg·kg^−1^ (ECR:Soil = 25:75, ECR size = 4.75–10.0 mm, and Ɵ_m_ = 35.5%) in Figure 4 for 180 days, respectively. Compared to the government standards of arsenic for the concerned (25 mg·kg^−1^) and the measurable (75 mg·kg^−1^) soil contamination in zone 1, the amount of released As was above the concerned standard (25 mg·kg^−1^) except ECR with mixing ratio (25:75) and particle size (4.75–10.0 mm). However, the released As from ECR in the soil solution may be dispersed into the soil by dispersion and diffusion depending on the water flow characteristics, and leached out as precipitate formation, such as scorodite at lower pH followed by precipitation of arsenical ferrihydrite at higher pH [24], so that the concentration of As in soil may become lower than the standards of government. The decrease in As released from ECR was more pronounced with increasing ECR particle size and ECR to soil mixing ratio (100:0) as shown in Figure 1. The amount of As released was more pronounced in Ɵ_m_ at 27% than those of saturation and 15% of Ɵ_m_ of all observations in 180 days. The amount of As released at 15% Ɵm for each sampling day was much less than the amount of As released at 27% and saturation. In particular, the amount of As release under 27% Ɵ_m_ was higher than that observed in the saturated condition throughout the experiment for all treatments, and the difference of released As between 27% Ɵ_m_ and saturation slightly increased with incubation time for all treatments. For this, we could conclude that the higher air content at 27% Ɵ_m_ attributed to more release of As from ECR. Based on these results, we could assume that As release could be influenced by the particle size of ECR, water content, and air content in this experiment.

## 4. Conclusions

The content of arsenopyrite which is the dominant As-containing sulfide mineral in ECR decreased with increasing particle size of ECR. The increase in ECR particle size, which is accompanied by a decrease in ECR surface area, results in a decrease in the amount of As released from ECR particles mixed with soil. The amount of As released from ECR mixed with soil could be influenced by the oxidation state depending on the water content of different sizes of ECR particles mixed with soil because the volume of the air-filled pore decreased with increasing mass water content for the same mixing ratio, while the volume of the air-filled pore increased with decreasing mixing ratio. The amount of released As from ECR mixed with soil was greater under unsaturated conditions near field capacity than saturated or close to wilting point conditions regardless of ECR particle size. The results can be interpreted in terms of the change of theoretical solubility of As due to the difference of Ɵ_m_ and air-filled pore volume for any given pH. The amount of As released from most of ECRs mixed with soil exceeds the relevant government concerned standard. The As released to the soil may disperse under dynamic water flow conditions and by diffusion due to differences in soil water potential as well as arsenic sulfide precipitation may occur under highly reduced conditions and with the presence of sulfide. Due to these phenomena, it is estimated that the actual arsenic concentration in the soil will be lower than the government standard. Therefore, it needs further investigation to select the appropriate incorporation criteria of particle size and rate of ECR depending on the physical and hydrological properties of the soil in terms of the government-concerned standard on As.

## Figures and Tables

**Table 1:** Characteristics of four groups of ECR and soil sample.

Category		pH (1:5)	EC (dS m^−1^)	OM * (g kg^−1^)	CEC (cmol_c_ kg^−1^)	As (mg·kg^−1^)	Particle Size Distribution (%)			Soil Texture
							Sand	Silt	Clay	
ECR	0–0.053	7.68	0.17	ND	0.77	4161.3	-	86.9	13.1	Silt loam
(mm)	0.053–2	7.41	0.15	ND	0.59	3706.1	-	-	-	
	2–4.75	7.29	0.13	ND	0.39	3283.5	-	-	-	
	4.75–10	7.14	0.12	ND	0.28	3023.4	-	-	-	
Soil		5.95	1.35	2.13	6.24	<0.03	42.7	36.6	20.7	Loam

* ECR—excavated crushed rock; OM—organic matter; CEC—cation exchange capacity.

**Table 2:** Soil particle distribution of ECR grades mixed with soil at various ratios.

Mixing Ratio	ECR (<0.053 mm) + Soil					ECR (0.053–2.0 mm) + Soil					ECR (2.0 mm<) + Soil				
	C	Si	Sa	G	ST	C	Si	Sa	G	ST	C	Si	Sa	G	ST
100:0	13.1	86.9	0	0	SiL	0	100	0	0	S	0	0	0	100	-
75:25	14.9	74.4	10.7	0	SiL	5.18	9.2	85.7	0	LS	20.7	36.6	42.7	75	SiL
50:50	16.9	61.8	21.4	0	SiL	10.4	18.3	71.4	0	SL	20.7	36.6	42.7	50	SiL
25:75	18.8	49.2	32.0	0	SiL	15.5	27.5	57.0	0	SL	20.7	36.6	42.7	25	SiL

C—clay; Si—silt; Sa—sand; G—gravel; ST—soil texture; S—sand; SiL—silt loam; SL—sandy loam; LS—loamy sand.

**Table 3:** Average bulk density and calculated porosity of ECR mixed with soil at four different ratios of ECR to the soil.

ECR:Soil Ratio	Volume of Soil (cm^3^)				Bulk Density (g cm^−3^)				Porosity			
	Particle Size of ECR (mm)				Particle Size of ECR (mm)				Particle Size of ECR (mm)			
	0–0.053	0.053–2.0	2.0–4.75	4.75–10.0	0–0.053	0.053–2.0	2.0–4.75	4.75–10.0	0–0.053	0.053–2.0	2.0–4.75	4.75–10.0
100:0	209.8	208.3	205.5	204.1	1.430	1.440	1.460	1.470	0.472	0.469	0.461	0.458
75:25	214.7	213.5	211.3	210.2	1.398	1.405	1.420	1.428	0.482	0.480	0.474	0.471
50:50	219.8	219.0	217.4	216.6	1.365	1.370	1.380	1.385	0.491	0.489	0.485	0.483
25:75	225.1	224.7	223.9	223.5	1.333	1.335	1.340	1.343	0.501	0.500	0.498	0.497

**Table 4:** Mass water content (Ɵ_m_) at saturation for four particle grades of ECR mixed with soil at five ratios.

Mixing Ratio (%)		Particle Grade (mm)			
ECR *	Soil	0.0–0.053	0.053–2.0	2.0–4.75	4.75–10.0
100	0	32.2	31.7	31.1	30.7
75	25	33.7	33.3	32.8	32.5
50	50	35.3	35.0	34.6	34.4
25	75	36.9	36.8	36.6	36.4

* ECR—excavated crushed rock.

**Table 5:** Volumes of water- and air-filled pore for ECR mixed with soil at four different ratios of ECR to soil at three levels of mass water content (Ɵ_m_).

ECR:SoilRatio		Ɵ_m_ (%)											
		15				27				Saturation			
		Particle Size of ECR (mm)				Particle Size of ECR (mm)				Particle Size of ECR (mm)			
		0–0.053	0.053–2.0	2.0–4.75	4.75–10.0	0–0.053	0.053–2.0	2.0–4.75	4.75–10.0	0–0.053	0.053–2.0	2.0–4.75	4.75–10.0
100:0	A	45.0	45.0	45.0	45.0	81.0	81.0	81.0	81.0	99.1	97.6	94.8	93.4
	B	54.1	52.6	49.8	48.4	18.1	16.6	13.8	12.4	0.00	0.00	0.00	0.00
75:25	A	45.0	45.0	45.0	45.0	81.0	81.0	81.0	81.0	104.0	102.8	100.6	99.5
	B	59.0	57.8	55.6	54.5	23.0	21.8	19.6	18.5	0.00	0.00	0.00	0.00
50:50	A	45.0	45.0	45.0	45.0	81.0	81.0	81.0	81.0	109.1	108.3	106.7	105.9
	B	64.1	63.3	61.7	60.9	28.1	27.3	25.7	24.9	0.00	0.00	0.00	0.00
25:75	A	45.0	45.0	45.0	45.0	81.0	81.0	81.0	81.0	114.4	114.0	113.2	112.8
	B	69.4	69.0	68.2	67.8	33.4	33.0	32.2	31.8	0.00	0.00	0.00	0.00

A—water-filled pore volume (cm^3^); B—air-filled pore volume (cm^3^).

**Table 6:** pH, EC, CEC, and As content of four particle grades of ECR mixed with soil at four ratios.

ECR	Soil	pH (1:5 H_2_O)				EC (dS m^−1^)				CEC (cmol_(c)_ kg^−1^)				Content of As (mg·kg^−1^)			
(%)		A	B	C	D	A	B	C	D	A	B	C	D	A	B	C	D
100	0	7.68	7.41	7.29	7.14	0.17	0.15	0.13	0.12	0.77	0.59	0.39	0.28	907	985.1	1111.8	1239.4
25	75	7.25	7.05	6.96	6.84	0.47	0.45	0.44	0.43	2.14	2.00	0.39	1.77	680.3	738.8	833.9	929.5
50	50	6.82	6.68	6.62	6.55	0.76	0.75	0.74	0.74	3.51	3.42	0.39	3.26	453.5	492.5	555.9	619.7
25	75	6.38	6.32	6.29	6.25	1.06	1.05	1.05	1.04	4.87	4.83	0.39	4.75	226.8	246.3	278	309.8

A: 0–0.053 mm; B: 0.053–2.0 mm; C: 2.0–4.75 mm; D: 4.75–10.0 mm.

**Table 7:** Mineral components of ECR depending on particle grade.

ECR * Size (mm)	Types of Minerals (%)							
	Calcite	Scorodite	Garnet	Magnetite	Aresenopyrite	Pyrite	Other	Total
0.0~0.053	66.3	17.7	7.83	6.21	1.28	0.34	0.34	100
0.053~2.0	68.9	18.9	5.61	4.75	1.14	0.35	0.35	100
2.0~4.75	73.5	20.5	2.17	1.98	1.01	0.41	0.43	100
4.75~10.0	73.8	20.3	2.07	2.03	0.93	0.41	0.46	100

* ECR—excavated crushed rock.

**Table 8:** Chemical composition of ECR depending on sample size (unit in wt. %).

Composition (%)	ECR * Size (mm)			
	0–0.053	0.053–2.0	2.0–4.75	4.75–10
CaO	49.5	46.3	39.8	35.5
SiO_2_	22.2	25.9	28.9	30.1
Fe_2_O_3_	17.3	16.9	19.9	22.6
MgO	4.07	4.18	4.42	4.82
Al_2_O_3_	2.67	2.56	2.57	2.51
As_2_O_3_	1.39	1.36	1.25	1.13
MnO	1.51	1.38	1.34	1.43
SO_3_	0.42	0.44	0.64	0.63
WO_3_	0.21	0.21	0.28	0.32
K_2_O	0.19	0.20	0.17	0.21
TiO_2_	0.17	0.15	0.15	0.11
ZnO	0.14	0.15	0.18	0.19
P_2_O_5_	0.11	0.17	0.17	0.18
CuO	0.12	0.10	0.15	0.17
Co_2_O_3_	ND	0.00	0.08	0.12
Total	100.0	100.0	100.0	100.0

* ECR—excavated crushed rock.

**Table 9:** Chemical characterization of arsenopyrite contained in ECR *.

Sample	Weight (%)						Atomic (%)		
	As	Fe	S	Co	Bi	Total	As	Fe	S
Arsenopyrite	45.6	32.4	21.2	0.02	<0.01	99.22	32.5	31.5	35.1

* ECR—excavated crushed rock.

## Data Availability

The data presented in this study are available on request from the corresponding author.
